# An evolutionary perspective on signaling peptides: toxic peptides are selected to provide information regarding the processing of the propeptide, which represents the phenotypic state of the signaling cell

**DOI:** 10.12688/f1000research.6874.1

**Published:** 2015-08-07

**Authors:** Keith Daniel Harris, Ari Barzilai, Amotz Zahavi

**Affiliations:** 1 Department of Zoology, Tel-Aviv University, Tel Aviv, 69978, Israel; 2 Department of Neurobiology, Tel Aviv University, Tel Aviv, 69978, Israel; 3 Sagol School of Neuroscience, Tel Aviv, 69978, Israel

**Keywords:** Signaling peptides, handicap principle, signal selection, evolution

## Abstract

Structurally similar short peptides often serve as signals in diverse signaling systems. Similar peptides affect diverse physiological pathways in different species or even within the same organism. Assuming that signals provide information, and that this information is tested by the structure of the signal, it is curious that highly similar signaling peptides appear to provide information relevant to very different metabolic processes. Here we suggest a solution to this problem: the synthesis of the propeptide, and its post-translational modifications that are required for its cleavage and the production of the mature peptide, provide information on the phenotypic state of the signaling cell. The mature peptide, due to its chemical properties which render it harmful, serves as a stimulant that forces cells to respond to this information. To support this suggestion, we present cases of signaling peptides in which the sequence and structure of the mature peptide is similar yet provides diverse information. The sequence of the propeptide and its post-translational modifications, which represent the phenotypic state of the signaling cell, determine the quantity and specificity of the information. We also speculate on the evolution of signaling peptides. We hope that this perspective will encourage researchers to reevaluate pathological conditions in which the synthesis of the mature peptide is abnormal.

## Introduction

**Figure 1.**
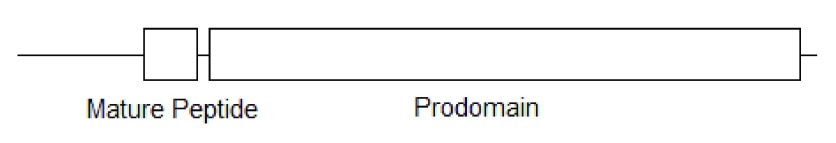
The organization of the prodomain and mature form in the propeptide of GnRH.

Signaling peptides are amino acid chains with diverse structures that serve as signaling molecules. The lengths of signaling peptides vary greatly from less than ten amino acids (such as oxytocin and vasopressin) to over 100 amino acids (such as the neurotrophic factors). The mature signaling peptide which is secreted is processed from a longer propeptide which contains other domains (prodomains) which are not part of the mature form ( Figure 1). 

Mature peptides of similar structure may function as a neurotransmitter, an endocrine or a paracrine signal within a multicellular organism, and also as a signal between unicellular organisms. For instance, the gonadotropin-releasing hormone (GnRH), which has a significant structural similarity to the yeast mating factor-alpha peptide ^1^, serves as both a hormone in mammals and as a mating pheromone in yeast ^1^. It also serves as a paracrine signal in the periphery of the multicellular organism ^2^.

Thus, though the structure of the mature signal of signaling peptides such as GnRH is conserved, its specific signaling role is not, and their prodomains differ markedly. Moreover, in the same organism, structurally similar signaling peptides may regulate a diverse range of signaling pathways, such as the structurally similar oxytocin and vasopressin ^3^, which also function as signals in unicellular organisms ^4^.

Assuming that signals elicit a response because they provide specific information that benefits the organism ^5, 6^, how may similar peptides provide information regarding such diverse metabolic processes?

While the mature peptides of GnRH, oxytocin and vasopressin are short (9-10 amino acids), their propeptides are large proteins (100-160 amino acids). The cleavage of the propeptide to form the comparatively short mature peptide is often dependent on the completion of post-translational modifications, such as sequential enzymatic modification ^7^, glycosylation, glycosulfation or the pairing of S-S bonds ^8^.

As the cleavage of the mature peptide depends on the propeptide completing its various post-translational modifications, and as there is a fixed stochastic relationship between the mature peptide and the propeptide that is determined by the number of repeats of the mature domain within the propeptide, it is reasonable to assume that the ability of the signaling cell to complete the synthesis of the propeptide is the information provided by the mature peptide.

We suggest that while the synthesis and modifications of the propeptide are related to the phenotypic state of the signaling cell, the role of the mature peptide is to stimulate cells to be attentive to this information. In this opinion paper we briefly review a number of signaling peptides to support our suggestion and, in addition, speculate why and how a mature peptide is selected to serve as a stimulating molecule.

## Different propeptides produce similar mature peptides

**Figure 2.**
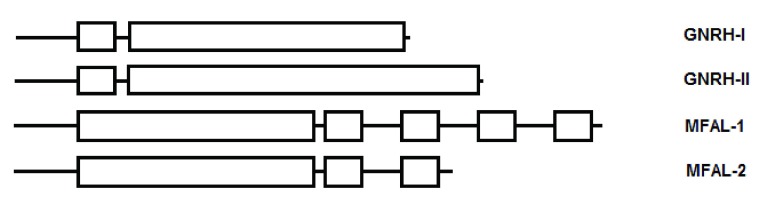
Schematic representation of GnRH and the yeast genes that encode mating factor alpha. Small boxes represent the mature domain.

Similar signaling peptides are used in different species to affect diverse metabolic processes; however, in many cases these similar peptides are processed from entirely different propeptides. Such is the case of the 10-amino acid GnRH, a hormone produced by the hypothalamus and also by cells in the periphery in vertebrates, which is structurally similar to the mature peptide of the yeast mating-alpha factor ^1^. Also within the yeast genome, a similar mature peptide (mating-alpha factor) is produced by two different propeptides encoded by the genes *MFAL-1* and *MFAL-2*. *MFAL-1* has four tandem repeats of the mature domain, while *MFAL-2* contains two repeats of the mature domain with a slight variation in sequence ( Figure 2).

**Figure 3.**
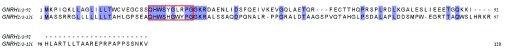
The similarity of GnRH and variation in the prodomain of the human genes encoding GnRH. Blue-filled boxes indicate high conservation. Red box contains the mature peptide sequences.

Even in the same species, the structurally similar forms of the GnRH peptide are connected to different prodomains. There are two genes in humans that encode the sequence of GnRH: *GNRH-I* and *GNRH-II*. Each gene has a highly similar mature peptide, but a different prodomain ( Figure 3). The synthesis of the mature form (GnRH) requires an intricate series of enzymatic modifications ^7^.

**Figure 4.**
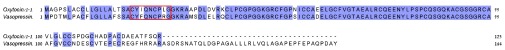
The similarity between oxytocin and vasopressin. Blue-filled boxes indicate high conservation. Red box contains the mature peptide sequences.

Oxytocin and vasopressin are structurally similar peptides of nine amino acids. The mature peptides of oxytocin and vasopressin are highly similar, and they share an accessory protein, neurophysin, yet the other domains have little similarity in sequence ( Figure 4).

The mature peptides of oxytocin and vasopressin are also active in unicellular organisms ^4^. When comparing oxytocin and vasopressin propeptides across the phylogenetic tree, it is evident that the mature domain is more conserved than the prodomain ^12^.

**Figure 5.**
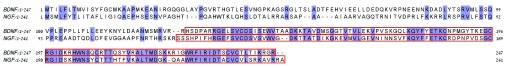
Sequence similarity between human BDNF and NGF. Blue-filled boxes indicate high conservation. Red boxes contain the respective mature peptide sequences.

**Figure 6.**
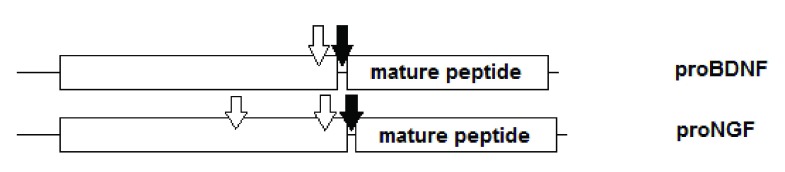
Structural similarity between the propeptides of the neurotrophic factors. Filled arrows denote cleavage site between prodomain and mature domain, empty arrows denote glycosylation sites.

In addition, within the same species, within a conserved family of signal peptides, such as the neurotrophic factors BDNF, NT-3 and NGF, the variation of the sequence of the mature peptide between the different peptides is significantly lower than the variation among the prodomains of the respective propeptides ( Figure 5). The variation in glycosylation sites is depicted schematically in Figure 6. This variation is also evolutionarily conserved: the sequence of the prodomain is unique while the sequence of the mature peptide is common to the family.

## The phenotypic state of the signaling cell affects the synthesis of the mature peptide

This variation is not only genetic, but may also be phenotypic. In the case of BDNF, there are several alternative transcripts that produce isoforms of BDNF. These isoforms differ in their prodomain but not in their mature domain, and this variation depends on metabolism and physiological parameters of the signaling cell. The production of specific isoforms of BDNF has been correlated to various pathological conditions in which the synthesis of BDNF is altered ^13^.

BDNF, NGF and NT-3 each have a conserved N-glycosylation site in the prodomain that is proximal to the processing site at which the propeptide is cleaved to form the mature peptide ^8^. When N-glycosylation is blocked, cleavage of the propeptide is affected, and less of the mature form is synthesized. What accumulate in the Golgi are truncated forms of proBDNF ^8^. The secreted mature form of BDNF is therefore a representation of the properly processed and cleaved proBDNF.

## Propeptide non-signaling functions

We know of two cases in which a whole functional protein that has a non-signaling role serves as the propeptide of a mature peptide signal. One of them is the sex pheromone system of *Enterococcus faecalis* ^14^, the other the extracellular death factor (EDF) of *Escherichia coli* ^15^.

As far as we are aware, there is no known non-signaling function for the propeptides of the neurotrophic factors. Likewise is the case for oxytocin and vasopressin, and also GnRH. However, cases in which a non-signaling function is known may illustrate how peptide signaling systems evolved.

*E. faecalis* is a bacterium that has a sophisticated mechanism of plasmid transfer governed by signaling peptides of 7-8 amino acids in length. These peptides are produced from specific membranal proteins that perform non-signaling functions in the cell that are unrelated to the plasmid ( Table 1). The mature peptide of the *E. faecalis* pheromones is part of the sequence of the propeptide which anchors it to the membrane. The cleavage of the propeptide from the membrane releases the mature peptide (the pheromone), which provides via a complex transduction mechanism ^14^ reliable information that the signaling cell does not possess the plasmid.

**Table 1.** Enterococcus pheromones and the function of their propeptides ^16^.

Plasmid function	Pheromone primary structure	Propeptide function
pAD1 – Haemolysin/bacteriocin and UV resistance	LFSLVLAG	Membrane immunogen with FMN-binding domain*
pCF10 – Tetracycline resistance	LVTLVFV	YidC membrane insertase
pPD1 – Bacteriocin	FLVMFLSG	YidC membrane insertase
pAM373	AIFILAS	Peptidase
pOB1 – haemolysin/bacteriocin	VAVLVLGA	ABC Methionine transporter substrate-binding protein

* Based on similarity

Another example of a signaling peptide whose propeptide serves a non-signaling function in the cell is the extracellular death factor (EDF) pentapeptide that activates the mazEF pathway in *E. coli*. The sequence of the pentapeptide is NNWNN, and it is synthesized by the proteolytic cleavage of the enzyme glucose-6-phosphate dehydrogenase ^17^, which includes the sequence NNWDN. The mature peptide requires the modification of the aspartic acid in the propeptide to asparagine.

As the propeptide of the pentapeptide is a functional protein, the relationship between its synthesis and degradation links the production of the pentapeptide directly to cell metabolism, and specifically the metabolism of glucose. Since the pentapeptide appears in the *E. coli* genome only within this enzyme, and is an essential component of the enzyme, its secretion from *E. coli* is reliable information that the signaling bacterium does not need the enzyme for its current metabolism.

## Discussion

The main purpose of this article is to propose a solution to the problem we faced when trying to understand how very similar short peptides may provide information that is relevant to receiver cells designed to serve very different roles. Mature peptides are often conserved across the phylogenetic tree, from unicellular organisms to mammals. Hence, it is tempting to attempt to identify what properties of these mature peptides cause them to be adapted for their role. Our perspective is derived from the assumption that signals provide reliable information regarding the behavior of the signaling cell ^6, 18^.

**Figure 7.**
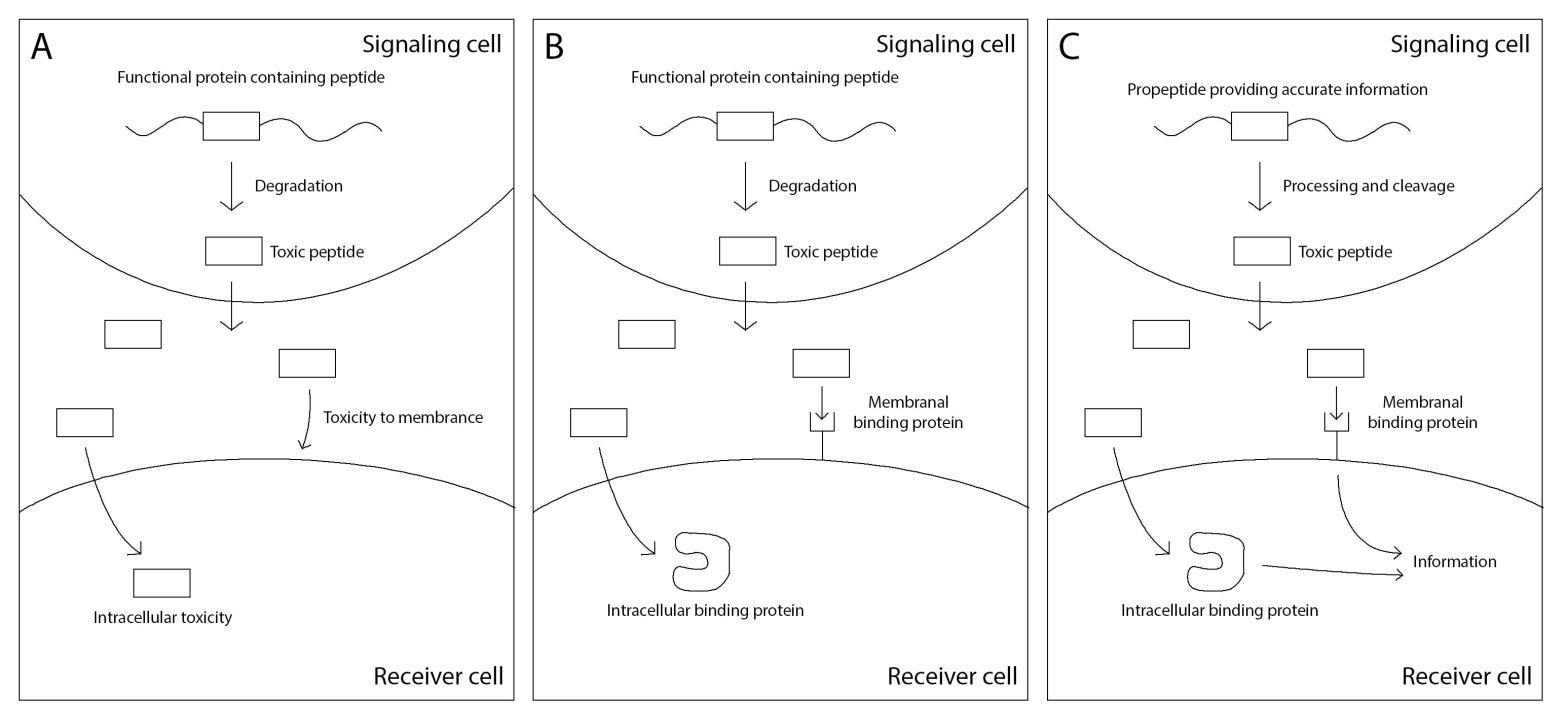
Toxic short peptides that harm neighboring cells may evolve into signals that provide information regarding the activity of the signaling cell. **A** A functional protein is degraded to produce short peptides, one of which is toxic and is secreted. The toxic peptide harms neighboring cells by interfering with the cell membrane or diffusing into the cell and interfering with intracellular processes. **B** Mechanisms evolve that counter the toxicity of the peptide, such as binding proteins on the cell membrane or in the cytoplasm. **C** The interaction between the secreted toxic peptide and the binding proteins may further evolve into a signaling system, as the secretion of the toxic peptide reflects the phenotypic state of the secreting cell.

We suggest that mature peptides were selected as optimal carriers for transferring information due to their ability to stimulate the receiving cell to attend to the information they represent. We speculate that their advantage as stimulating agents is due to their harmful effects which force the receiver to attend to the information ( Figure 7).

## Predictions

Our suggestion that the mature peptide does not directly represent information regarding the signaling cell's metabolism, but reflects information regarding the synthesis of the prodomain, can be tested. Here we suggest experiments that may test this suggestion:

1. Replacing the mature peptide of one propeptide with a similar mature peptide from the same family of peptides (such as oxytocin with vasopressin), may reveal that the mature peptide has the same effect due to its specificity for its receptor; however, we expect that the timing and quantity of the effect will be determined by the protein to which it is attached.
2. The harmful effects of the mature peptides may be assessed by increasing their quantities beyond the ability of the receiving cells to counter their noxious effects to which they are usually exposed or by removing any other mechanisms that under normal circumstances counter these harmful effects, such as enzymes that degrade the mature peptide or bind it.

## Some speculations on the stages of the evolution of signal peptides

It is tempting to speculate how the sequence of mature peptides evolved, even though at present we have succeeded in collecting limited data to support this speculation.

It is reasonable to assume that the first generation of mature peptides were part of large proteins that had non-signaling functions in the cell. During the proteolysis of these large functional proteins, short peptides were secreted. Among the short peptides cleaved and secreted from cells, the peptides that harmed neighboring cells selected, in neighboring cells, for mechanisms that counter the harmful effects of the peptides. The level of the response to the harmful effects is correlated to the level of the secreted peptide and, hence, could be used by the neighboring cells as a source of information regarding the metabolism of the proteins from which they were cleaved in the signaling cells. These large proteins still serve their initial non-signaling function, yet they also serve as propeptides. These are the cases mentioned previously of the EDF pentapeptide of *E. coli* which is a functional element of glucose-6-phosphate dehydrogenase ^16^ and the enterococcus pheromones, which anchor the propeptide to the membrane ^17^.

We suggest that a signal which harms the receiver will force the receiver to respond to a smaller change in its concentration than a signal that provides a positive or neutral effect. Zahavi ^19^ and Zahavi & Zahavi ^5^ found this to be the case for many signals used by birds and humans. In addition, Harris *et al.* ^20^ pointed out that several non-peptide signals such as glutamate and dopamine may cause harm to cells that do not counter their harmful effects in relation to their level of release. They suggested that the toxicity ensures that the response of the receiver cell is correlated to the level of the release from the signaling cell.

We also suggest that in the case of signaling peptides, the toxicity of the mature peptide ensures that the response of the receiving cell is correlated to the concentration of the secreted peptide. At present we are aware of only one case in which a peptide has a known direct toxicity that is crucial to its function, the EDF pentapeptide. The pentapeptide kills *E. coli* by binding to the mazF toxin and interfering with the ability of the mazE antitoxin protein to inhibit the activity of the mazF toxin ^15^.

**Figure 8.**
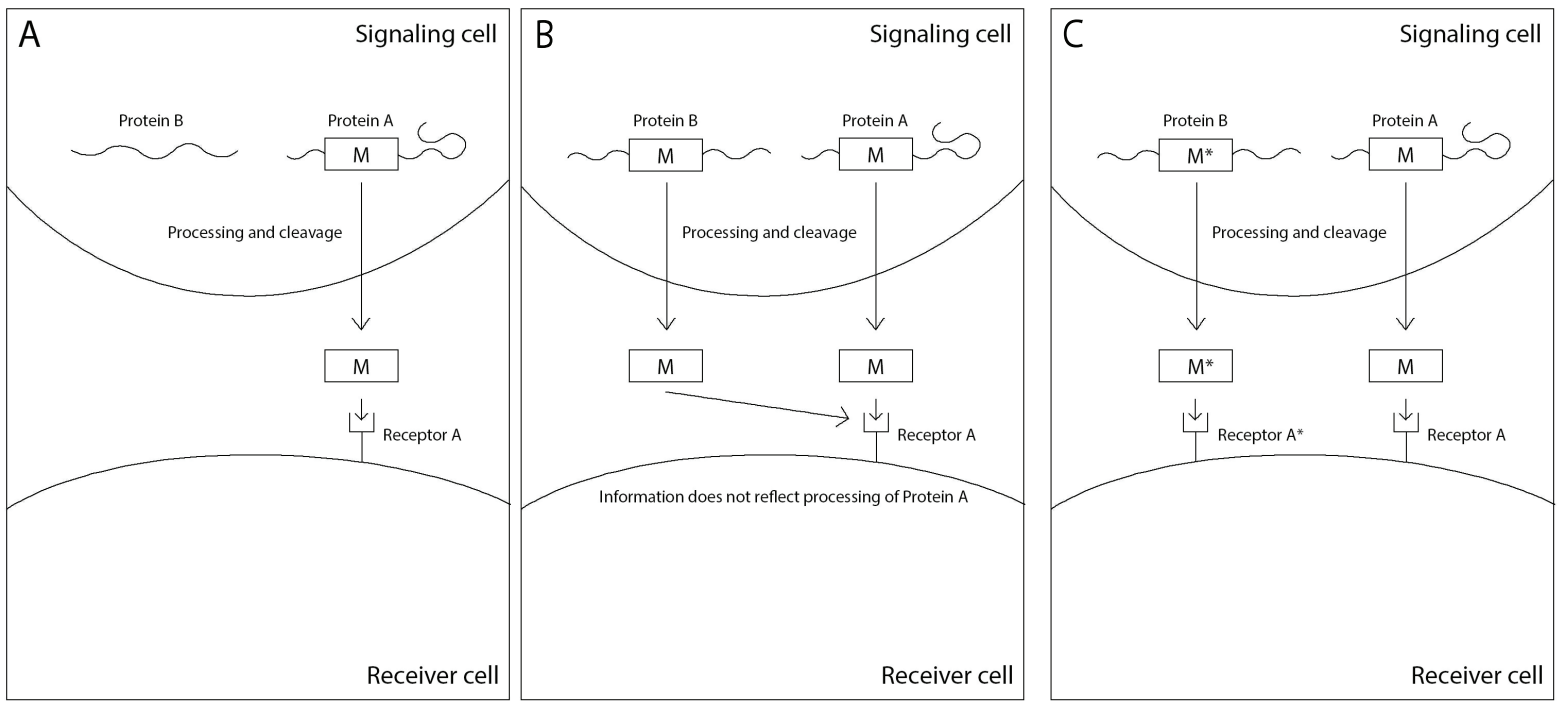
Mutations may transfer mature toxic peptides to other functional proteins and evolve new signals. **A** A function protein (Protein A) contains a toxic signaling peptide (M) which has a complementary receptor (Receptor A) **B** A mutation causes the toxic signaling peptide M to be transferred into a different functional protein (Protein B), so that both proteins produce the same toxic peptide that interacts with Receptor A. The toxic peptide M no longer provides accurate information regarding the processing of Protein A. **C** A mutation in the toxic peptide M in Protein B (yielding the toxic peptide M*) prevents its binding to the same receptor as M, and a receptor specific to M* (Receptor A*) evolves to counter its toxicity, which may provide information on the processing of Protein B.

Once the sequences of mature peptides evolved as carriers of information due to their ability to stimulate cells to attend to information, natural selection could transpose a mature peptide to be a part of other proteins whose synthesis reflected information regarding the phenotypic state of the signaling cell, granted that this information benefitted the organism through its effect on the receiving cells. Small modifications in the mature peptide of the first generation were required to prevent the binding of the new mature peptide to the receptors of the first generation peptides, if the first generation and the second generation mature peptide were to function in the same organism ( Figure 8).

The third generation of mature peptides is secreted from central glands and from the brain to activate peripheral cells. Wessler *et al.* ^21^ suggested that acetylcholine, which serves as a paracrine signal to coordinate activities between epithelial cells in the airpipe, evolved first as a paracrine signal and was only later adopted by the brain to activate peripheral cells that already respond to acetylcholine. Following this suggestion, we recently proposed a general model for the evolution of non-peptide signals ^20^. It is reasonable to assume that signals which function between cells in the periphery, and are also used by the brain and central glands for their similar effect on peripheral cells, evolved first as paracrine signals in the periphery ^18, 21^ (i.e., neurons and endocrine cells adopted the mature peptides that served as signals in the periphery to activate cells that were already adapted to respond to them).

In several cases it is known that the mature peptide by which the brain stimulates peripheral cells is also synthesized in small quantities by the cells that respond to it. Oxytocin and GnRH for example are synthesized in peripheral cells that respond to the same peptides that are secreted in the brain. The present function of the synthesis of the small amounts of mature peptides in peripheral cells is unclear.

It is reasonable to assume that the function of the propeptides in the brain is not to provide information relating to the metabolism of the secreting neuron, but to allow the synthesis of the mature peptide in large quantities to regulate and synchronize the activities of various organs to respond to decisions made in the brain. Hence, we expect that the structure of the propeptides may differ between the brain and peripheral cells, as each serves a different need: neurons need to synthesize large quantities of the mature peptide, while in the periphery the propeptide reflects the phenotypic state of the signaling cell.

## How our perspective changes the focus of treatments for pathological conditions in which the mature peptide is lacking

Many neuropathologies, including amyotrophic lateral sclerosis, Alzheimer's diseases and Parkinson's disease, have been associated with a reduction in the synthesis of neurotrophic factors ^22– 24^. Current treatments of these diseases often involve administering synthesized mature peptides (the neurotrophic factors). However, if the inability to process the propeptide correctly (such as the inability to perform the glycosylations) reflects reliably the phenotypic state of the signaling cell, then these treatments provide false information to the neuron. The false information may mask the underlying problem, which may be the deterioration of the signaling cell’s ability to serve its function. If the mature peptide provides retrograde information to the neuron on the ability of the peripheral cell to be activated by the neuron, then supplementing it may mislead the organism that the system is functioning correctly even though it is not. In addition, if, as we suggest, the mature peptide functions due to its harmful effects, then providing an abnormal concentration of it to the receiver cells may cause unnecessary damage to the signaling system. Hence, the attempts to counter the disease should focus on attempting to improve the phenotypic state of the signaling cells, for instance, by providing anti-oxidants ^25^.

## Methods

All sequences were taken from the UniProt database ^9^. Alignments were calculated using Clustal Omega ^10^ implemented in UniProt ^9^. Jalview v2.8.2 ^11^ was used for the graphical representation of the alignments.
