# Supplementary material for: An evolutionary perspective on signaling peptides: toxic peptides are selected to provide information regarding the processing of the propeptide, which represents the phenotypic state of the signaling cell
Source: F1000Res. 2015 Aug 7;4:512. [Version 1] doi: 10.12688/f1000research.6874.1 (PMC4648229; doi:10.12688/f1000research.6874.1)
Supplement: Peer review history

# Peer review: Referee response for version 1

**DOI:** 10.5256/f1000research.7401.r10773

**Published:** 2015-10-12

**Authors:** David Granot

**Affiliations:** 1 Department of Vegetable Research, Institute of Plant Sciences, Agricultural Research Organization, Volcani Centre, Bet Dagan, Israel

This opinion article addresses basic questions about the nature of signals in biological systems. The underlying assumption is the brilliant handicap theory of Amotz Zahavi that signals must have some burden on the signaling cell to be perceived as a reliable signal.

The central question that this opinion article is dealing with is how very similar (mature) signaling peptides control different metabolic pathways in different species or even within the same organism.

The authors suggest that the message (signal) resides in the propeptides that yield the mature signaling peptides. The amount, location and timing of the very similar (yet not identical) signals provide a reliable and specific information on the capability of the producing cells to synthesize, modify, cleave and secrete the mature signaling peptides.

They also suggest that the mature signaling peptides might have toxic effects on the receiving cells that enforce them to respond.

The authors also offer an evolutionary prediction of how such signaling peptides might have evolved from propeptides that might have functions which are not necessarily related to the signal. Finally they offer a perspective on the potential impact of their theory on evaluation of pathological treatments, in which the current practice is increasing the amount of the signaling peptides rather than dealing with malfunction of the source of the peptides.

As usual, the theories stemming from Zahavi’s and his colleagues (Harris and Barzilai this time) are stimulating, but require validation, as emphasized by the authors themselves. Without validation, the appeal of theories is dependent on how reasonable they are, and if they make (new) sense of disturbing questions. The suggested theories should also be evaluated in comparison with alternative theories. It is not clear if alternative theories exist, and if they do, I would suggest discussing them.

The major question raised by the authors is how very similar signaling peptides have different functions?

Although very similar, even small signaling peptides are not identical, and it is advised not to exclude the option that the specificity resides within the different amino acids. The examples brought by the authors of cross species effects of similar peptides support the authors’ theory, but can also be explained by the modular nature of biological structures of signaling peptides and receptors. As suggested by the authors, shuffling mature peptides between propeptides may provide an answer.

An important part of the author’s hypothesis is that signals might (should?) be toxic to the receiving cell to enforce a response. The only example is the *E. coli *EDF pentapeptide that has a direct toxicity that is crucial to its function.

The biological logic of toxicity to the receiving cells as a way to enforce response is not clear. Toxicity means that the outcome could be harmful, such as killing or deactivating the receiving cells. Do the authors suggest that in such cases the signaling peptide has also a selection function?

The authors also discuss a case in which neurons within the brain produce a signaling peptide that affects peripheral cells and suggest that although processed from propeptides in the brain it does not provide information relating to the phenotype of the secreting neuron.

It is not clear why these neurons are excluded from the “handicap principle”? Furthermore, dealing with pathological conditions related to the administration of overdose of the signaling peptides (in Alzheimer disease), the authors suggest that it might mask the “inability to process the propeptide correctly by the signaling cell” which could be the cause of the disease. Wouldn’t that mean that production of signaling peptide from the propeptide in neurons should provide information about the phenotype of the secreting neuron?

As hinted above, brain stimulation by various scientific hypotheses is usually positive, and may indicate the excellent “phenotypic state” of the current “signaling” group of authors. Yet, I hope there is some “toxicity” that may enforce few readers to respond and test the hypotheses

I have read this submission. I believe that I have an appropriate level of expertise to confirm that it is of an acceptable scientific standard.

# Peer review: Referee response for version 1

**DOI:** 10.5256/f1000research.7401.r10173

**Published:** 2015-09-01

**Authors:** Uzi Motro

**Affiliations:** 1 Department of Ecology, Evolution and Behavior, The Hebrew University of Jerusalem, Jerusalem, Israel

A stimulating paper, suggesting a new perspective on the nature of signaling peptides.

The main idea can be summarized as follows:

1. The mere structure of mature signaling peptides does not necessarily convey the information they carry, but rather their sheer existence, which is due to *successful* processes undergone by their propeptides, reliably indicates the phenotypic state of the signaling cell.
2. The information given by signaling peptides is heard by the receiving cell because of the toxic nature of the signaling peptides – their toxicity cannot be ignored by the receiving cell.

This is a truly novel idea, which seems to be a solid consequence of natural selection. The authors present a few examples, yet – as they point out – sound experimental evidence is necessary. As a theoretician of evolutionary biology (but by no means an expert in cell biology!) I am not in a position to comment or to suggest such an experimentation. If this idea is valid, the consequences to some medical treatments can be substantial, as indicated in the paper's last section.

I have read this submission. I believe that I have an appropriate level of expertise to confirm that it is of an acceptable scientific standard.
